# Equine Melanocytic Tumors: A Narrative Review

**DOI:** 10.3390/ani13020247

**Published:** 2023-01-10

**Authors:** José Pimenta, Justina Prada, Mário Cotovio

**Affiliations:** 1 Veterinary Sciencies Department, University of Trás-os-Montes e Alto Douro, 5000-801 Vila Real, Portugal; 2 CECAV-Veterinary and Animal Research Center, University of Trás-os-Montes e Alto Douro, 5000-801 Vila Real, Portugal; 3 Associate Laboratory for Animal and Veterinary Sciences (AL4AnimalS), University of Trás-os-Montes e Alto Douro, 5000-801 Vila Real, Portugal

**Keywords:** equine, melanocytic tumors, cutaneous neoplasia

## Abstract

**Simple Summary:**

Melanocytic tumors are among the most common cutaneous neoplasms in horses. Contrary to popular opinion, the slow rate of growth typical of these tumors does not warrant benign classification. Furthermore, the wide spectrum of treatment modalities can generate some frustration for equine practitioners. This narrative review aims to give an overview of the genetic basis, linked with melanoma development, of alterations in STX17, ASIP and MITF genes, a description of the clinical and pathological differences between naevus, dermal melanoma, dermal melanomatosis and anaplastic malignant melanoma, as well as a description of the role of the different diagnostic tools, mainly fine needle aspiration, histopathology and immunohistochemistry. The most common treatment modalities, such as surgery, chemotherapy and electroporation are reviewed, and a description of less common options, such as immunotherapy and radiotherapy is presented. The article also elucidates future perspectives and research fields about equine melanocytic tumors, mainly in the search for new therapeutical targets, such as CD47, PD-1 and COX-2.

**Abstract:**

Adult grey horses have a high incidence of melanocytic tumors. This article narratively reviews the role of some genetic features related to melanoma formation in horses, such as STX17 mutation, ASIP or MITF alterations, and the link between the graying process and the development of these tumors. A clear system of clinical and pathological classification of melanocytic tumors in naevus, dermal melanoma, dermal melanomatosis and anaplastic malignant melanoma is provided. Clinical and laboratorial methods of diagnosing are listed, with fine needle aspiration and histopathology being the most relevant. Relevance is given to immunohistochemistry, describing potentially important diagnostic biomarkers such as RACK1 and PNL2. Different therapeutical options available for equine practitioners are mentioned, with surgery, chemotherapy and electroporation being the most common. This article also elucidatesnew fields of research, perspectives, and new therapeutic targets, such as CD47, PD-1 and COX-2 biomarkers.

## 1. Introduction

Equine melanocytic tumors have been a great cause of concern to owners and veterinarians due to the economic and health consequences of their presence [1]. Many authors agree that every grey horse will develop a melanocytic tumor if left long enough, as there is an age-related predisposition [2].

Approximately 80% of equine tumors involve the skin, of which 15% are melanocytic. More than 90% of equine melanocytic tumors are benign; however, around 66% tend to progress to malignant forms, being able to metastasize [1,3,4]. Although melanocytic tumors have been diagnosed in horses of all coat colors, grey horses seem to be especially predisposed, with an 80% prevalence in animals older than 15 years [5,6]. Non-grey horses such as bays and chestnuts could also have melanocytic tumors which are more likely to be malignant and have early metastasis; this is probably because they experience a higher activity of melanocytes compared with grey horses [5].

Melanocytic tumors result from abnormal proliferation of melanocytes [7]. Melanocytes are dendritic cells derived from neuroectodermal melanoblasts mainly found in the skin, within the basal layer of the epidermis interspersed between basal keratinocytes and in the outer root sheath of hair follicles [1,2]. The normal melanocyte is isolated from other melanocytes and does not have melanin retained inside. Instead, melanin is packaged in melanosomes and passes through dendritic processes to keratinocytes [1]. Through melanogenesis, these cells produce melanin that will be present in the skin, eyes and hair. Because of its dark color, melanin can absorb UV-B light, protecting the skin from the dangerous effects of solar radiation [8].

The heritability of melanocytic tumors in grey horses has been shown to be moderate [9]. A study with 295 grey Lipizzaner horses reports a heritability estimate of 0.36 with a standard error of 0.11, demonstrating the impact that additive inheritance may have on the development of disease [10]. Although some breeds seem to be genetically predisposed, such as Arabians, Lipizzaner, Thoroughbred and Andalusian horses, it is not clear if this predisposition is breed related or influenced by the high prevalence of grey coat color in these breeds [1,10,11].

This narrative review describes the etiology, classification, clinical presentation, diagnostic work-up and current treatments of equine cutaneous melanocytic tumors.

## 2. Etiology

Although historically controversial, currently cutaneous melanocytic tumors are accepted as a neoplastic disease. Molecular studies have been performed to characterize the pathways that lead to neoplastic transformation of melanocytes and pigmentary disorders in grey horses [2,12,13].

The greying phenotype is an autosomal dominant genetic trait related to a progressive depigmentation of hair. Pielberg et al. [14] indicated that the grey coat phenotype was associated with a 4.6 kb duplication within the introp 6 of syntaxin-17 (STX17) gene. The grey-inducing mutation STX17 (G) is autosomal dominant for coat color, which means that any horse (black, bay or chestnut) with G at one or both alleles (G/G or G/g) will become grey [9]. The mutation region includes four genes: NR4A3 (nuclear receptor subfamily 4, group A, member 3), STX17 (syntaxin 17), 40TXNDC4 (thioredoxin domain-contain-40) and INVS (inversin). Melanocytic tumors of grey horses overexpress SXT17 and the neighboring gene NR4A3 [7,15].

The duplicated region contains a cis-acting melanocytic enhancer that is upregulated by duplication [9,16]. These duplicated regions also contain additional binding sites for microphthalmia-associated transcription factor (MITF), which results in increased melanocyte proliferation [13,16].

Agouti signaling protein (ASIP) and melanocortin-1 receptor (MC1R) are two genes thought to play a key role in melanocytic tumor development. MC1R enables production of the black hair pigment eumelanin by increasing MITF expression, whereas ASIP reduces MC1R activation, inhibiting black hair pigment production. In the presence of an ASIP loss-of-function mutation, this inhibition does not occur. When unrestricted MC1R production of eumelanin caused by ASIP mutation is added to enhanced melanocyte proliferation caused by STX17 mutation, the likelihood of developing melanocytic tumors increases [7,15].

It is thought that a link between the greying gene and phenotypic effects could be the increased dermal melanocytes’ proliferation [10,14,17]. In hair follicles, melanocytes are recruited from a finite pool of stem cells that are continuously depleted. Overproliferation of melanocytes probably prematurely ends the stem cell supply. This could lead to progressive depigmentation of the hair [14,16]. Dermal and epidermal skin melanocytes under overproliferation are prone to neoplastic transformation, which leads to melanocytic tumor formation [7]. However, the greying process and the development of melanocytic tumors occur at different speeds between grey horses. The homozygous form of this duplication generates a quicker greying and a more uniform white coat color in adult grey horses, and even a higher incidence of melanocytic tumors [14]. All these findings are the genetic link between the grey coat color and the higher incidence of melanocytic tumors in grey horses [11]. Although the link between grey phenotypic mutation and melanocytic tumor development is well established, the molecular mechanisms behind it remain unknown; it is also unknown if there are additional somatic mutations participating in melanomagenesis.

The extracellular signal-regulated kinase (ERK) pathway has an important role in melanogenesis, with most part of human melanocytic neoplasms presenting a deregulation of this pathway and high levels of activated ERK1/2 being found on these tumors [18]. In human melanocytic tumors, ERK activation is caused mainly by somatic mutations in BRAF, RAS, GNAQ, GNA11, GTPases and KIT. A less common cause is a decreased expression of the negative regulators of the pathway [19,20]. According to these facts, Jiang et al. in 2014 analyzed ERK involvement in equine melanocytic tumors. Their results demonstrated that the ERK pathway is activated in equine melanocytic tumors without the presence of somatic mutations previously found in human melanocytic tumors. A link between ERK pathway activation and negative regulators was not found either. All tumors analyzed expressed ERK1/2; however, a significantly higher expression was present in grey horse samples. In addition, high levels of ERK1/2 were found in the normal skin of grey horses before tumor growth, providing more features about the predispositions of these kind of horses. Although this study suggests that 4.6 kb duplication of STX17 gene is the mutation that causes ERK pathway activation, deeper studies will be required to understand the relationship between this mutation, ERK pathway activation and melanocytic tumors’ onset. Recently, next-generation sequencing studies of equine melanomas found some mutated genes in common with human and dog melanoma, such as NRAS, TP53, KIT and BRAF which could function as activating mutations in the ERK pathway [21].

Very recently, through a genome-wide association study, Druml et al. [20] also suggested that the *DPF3* gene may contribute to the regulation of melanomatosis in grey horses.

In contrast with humans, ultra-violet radiation does not seem to be a risk factor for tumor growth, since some preferential locations are protected from the sun. Furthermore, horses maintain their dark skin color despite hair depigmentation, which provides good protection against UV radiation. Nevertheless, a clinical commentary from Kanellis [22] 8 expresses the hypothesis of implementing some sun safety practices in horses with non-pigment skin regions, since these areas, as well hairless areas, absorb the highest doses of ultra-violet radiation, propitiating actinic damage which may make them prone to melanocytic tumor development. This author proposes the use of sensors called melanometers, which can measure horse epidermal melanin concentration and individual maximum safe radiation exposure. With these parameters, one can quantify the ultraviolet sensitivity of an individual horse. With this data, owners and clinicians can apply some sun safety practices according to depigmentation, hairlessness degree and geographical location.

## 3. Classification

The nomenclature and classification of melanocytic tumors is complex for both clinicians and pathologists. Broadly, these tumors can be classified as benign or malign. Benign, also called melanocytoma, could be congenital or acquired, and describes a well-differentiated cell unlikely to infiltrate surrounding tissues and metastasize. Malign, also called melanoma, characterizes a less differentiated cell that likely infiltrates surrounding tissues, presents vascular and/or lymphatic invasion and recurrence after surgical excision and is prone to metastasizing. Due to evidence of the malignant propensity of most melanocytic tumors, the general term melanoma is frequently used to classify both benign and malign masses, with equine malignant melanoma (EMM) being used to classify the truly malignant forms of the disease [2,4,23,24,25,26].

Four categories have been proposed, three of which havepotential malignant behavior: naevus; dermal melanoma; dermal melanomatosis and anaplastic malignant melanoma [3,27]. Four uncommon types of naevi are currently described in horses, showing similarities with human naevi subtypes: melanocytic naevus, intradermal common melanocytic naevus, cellular blue naevus and combined cellular blue naevus [28].

Naevus is not restricted to grey horses and typically occurs at young ages (until 6 years old), appearing in atypical anatomical places such as the neck, limbs, body trunk and face. Melanocytic naevus is a benign form of pigmented lesion that occurs in horses of 6 years or less, could be congenital and usually has epidermal involvement. The remaining types of naevus have dermal involvement and can appear between 2 and 20 years old [3,28,29]. Because of its limited epidermal or superficial dermal involvement and its clinical and histological benign behavior, only the melanocytic naevus is truly considered a benign type or melanocytoma [1,2,5].

Dermal melanomas are isolated and discrete lesions; they are typical of grey horses from young adulthood until older adulthood and refer to the presence of a few spherical isolated masses. They can be either benign or malign [1,29].

Dermal melanomatosis usually involves older horses, being characterized by the presence of multiple and often coalescent masses. The previous two are the commonest types of diagnosed melanocytic tumors in horses. Many authors classify them as different stages of a single type, since there seems to be a clinical continuum between the two types. Histologically, they are very similar but have different clinical presentations [2,30].

Anaplastic malignant melanoma is a rare category more frequent in geriatric and non-grey horses. It is very aggressive, and capable of leading to death within a few months. It has fast growth, and often there is a heterogeneous grey or pink-grey pattern on the masses’ surface [30,31].

Dermal melanoma, dermal melanomatosis and anaplastic malignant melanoma present deep dermal locations and are characterized by a propensity for malignant behavior, since a part of them exhibits surrounding tissue infiltration and wide spreading to other locations and organs. Inclusively, there are some reports of spinal cord compression after infiltration by a perineal and base tail tumor mass into vertebral bodies and the paravertebral musculature of the lumbosacral spine (Rodríguez et al., 1998). Due to this propensity for malignancy, the concept of equine malignant melanoma (EMM) to describe these types of tumors is widely accepted [4,25,26]. Moore et al., 2013 proposed four different clinical stages to classify EMM, summarized on Table 1.

## 4. Clinical Features

Melanocytic tumors often appear as black cutaneous nodules of various sizes, most of them in grey horses and usually between 6 and 7 years old, corresponding to the age when their coat color changes [1,5]. In the early stages, the majority are melanocytomas and have an extended period of slow and benign growth patterns. Malignancy is age-related, being more prevalent in older grey horses as time passes. A high proportion of initially benign melanocytic tumors have the potential to, with time, acquire an aggressive behavior, become malignant and metastasize. This justifies the importance of an early approach to diagnosis and therapeutic options; there is a lack of evidence that interventions on these early benign tumors propitiate the changeover to an aggressive and malignant form, unlike in other skin tumors [1,32]. It is important to keep in mind that, although most of these tumors are black in color, some are amelanotic. These forms are often more aggressive than others [32].

In the beginning, the disease starts with small solitary dermal tumors with a predilection for the ventral tail, the perianal region and the external genitalia. Eyelids and lips are also common sites. Atypical sites have been documented, such as the coronary band, foot, mammary gland, vertebral region, parotid salivary gland, guttural pouch, third eyelid, esophagus, cornea and nasopharynges [4,32,33,34,35,36,37].

Clinical signs are variable, depending on the location, size and character of the tumor. Small benign tumors usually do not have any kind of interference; however, some can interfere with the bridle or saddle. Larger masses could have serious obstructive implications on the gastrointestinal and urogenital tracts. Cosmetic and debilitating effects can become more obvious when the mass becomes ulcerated and infected (with the presence of black exudate and hemorrhage), despite the lack of evidence that this is an indicator of transformation to malignancy. Chronic blood loss can lead to regenerative anemia. In the worst cases, life-threatening signs could emerge in face of metastasis that could spread through hematogenous or lymphatic ways to lymph nodes, other cutaneous tissues and internal organs such as the spleen, liver or lungs [5,6]. A resume of melanocytic tumors features is presented on Table 2.

## 5. Diagnosis

Diagnosis should try to establish the likely pathological and clinical effects of a tumor so the clinician may analyze the whole picture and make a sensible judgement on treatment options and prognosis. The earlier the diagnosis, the better the chances of an effective response to treatment. Since many different therapies are described for different kinds of tumors and tumor types, a precise diagnosis is crucial [6,23,38,39].

Physical examination could be more than accurate for a presumptive diagnosis of a skin melanocytic tumor, mostly when a black mass is found in a preferential location on a grey horse. However, tumors can arise in atypical places and can be amelanotic, and it can be difficult to differentiate them from other skin tumors, making diagnosis difficult. Furthermore, it is prognostically valuable to confirm if a tumor takes a benign or a malignant form [3,32,38,40].

### 5.1. Clinical Diagnostic Procedures

Biopsy sampling of tissue is the gold standard and usually, the histopathologic exam provides a definitive diagnosis when the sample is representative, well collected and sent to the laboratory in appropriate conditions [5,38]. Although uncommon in melanocytic tumors, there are always some risks in this procedure as an exacerbation of tumor reaction can occur, and tumor cells could be spread locally or gain access to blood vessels, mainly in already aggressive and malignant forms [30].

Fine needle aspiration (FNA) can be performed, and most of the time, black pigment is obvious during aspiration, giving the clinician a very sensitive presumptive diagnosis [30].

In cases of perianal tumors, rectal palpation or ultrasound should always be done to check for internal masses that can interfere with defecation [27].

### 5.2. Laboratorial Diagnostic Procedures

#### 5.2.1. Hematology and Biochemistry

In most skin tumors cases, there is no significant change in the broad spectrum of measured parameters in routine laboratory profiles. At their limit, changes can occur due to a state of chronic disease and chronic blood loss, usually presenting normochromic or hypochromic, normocytic or microcytic anemia [23]. However, Conrado et al. [41] identified a neutrophilia with multiple circulating melanin-containing neutrophils in a horse with disseminated melanoma during complete blood count analysis. This finding reveals that minimum bloodwork data in horses with cutaneous melanocytic tumors and presenting systemic signs of disease could help in the diagnosis of melanoma metastasis.

A recent preliminary study investigated the proteomic profile of 25 horses with different stages of melanocytic tumors to identify serum proteins that help in the differentiation between disease stages [42]. The results were compared between normal and non-normal groups (mild and severe disease) according to differentially expressed protein analysis. Most proteins found participate in lipid metabolism and presented overexpression in mild and severe stages, including lipin 2, phosphoinositide phospholipase C, beta-carotene oxygenase 1, long-chain fatty acid proteins, 3 hydroxy-3-methylglutaryl coenzyme A synthase, and sphingomyelin phosphodiesterase 3. The results considered phospholipid phosphatase6 (PLPP6) and sodium/potassium-transporting ATPase subunit alpha (Na+/K+-ATPase), two proteins involved in lipid and energy metabolism, respectively, as potential proteins only expressed in the mild stage of melanocytic tumor. These findings support previous knowledge about the increased rate of lipidic metabolism on melanocytic tumor cells important for growing and metastatic processes [43].

#### 5.2.2. Histopathology

Melanocytic tumors cells usually contain variable amounts of finely granular melanin pigment. Often there are many melanophages with large amounts of coarse melanin within the cytoplasm and with peripheral and small nuclei [2]. When obscured by melanin, the visualization of tumor cell detail and mitotic rate is difficult. Bleaching with permanganate potassium is necessary to remove pigment and have a clear cytoplasm of these cells [2].

Although histopathology is a reference diagnostic tool, it has some limitations. Differentiation between benign and malign masses is not always accomplished; different types of the same tumor could be histologically very similar. In early stages, it could be difficult to separate early melanoma from melanocytes; moreover, with disease progression, and due to the loss of cell differentiation and minimal pigment content, it could be difficult to recognize its melanocytic origin and distinguish it from other tumors [27,44,45,46]. Here, we present a histological description of the equine melanocytic tumor types mentioned before.

##### Melanocytic Naevus

The main difference between melanocytic naevus and the three other types of naevi is epidermal involvement. It is composed of clusters of neoplastic melanocytes distributed through the superficial dermis and dermal–epidermal junction, with frequent epidermal involvement and numerous heavily pigmented melanophages between cells. These cells are polygonal, relatively large and can sometimes show features of malignancy such as high mitotic activity and pleomorphism [2,28].

##### Intradermal Common Melanocytic Naevus

Tumoral masses are usually raised, non-encapsulated, well-demarcated and symmetrical. They could be localized in the superficial and middle dermis or within the deep dermis. Some tumor cells could infiltrate in adnexal structures and vessels. The overlying epidermis may show segmental hyperplasia, hyperpigmentation, atrophy, erosion and ulceration. Areas of granulomatous inflammation can be found close to sebaceous glands.

Naevus cells of different shapes are clustered in multiple islands that have a pale and myxomatous stroma. Mild anisocytosis, anisokaryosis and cellular pleomorphism are present. The mitotic rate may vary from 0 to 8 mitotic figures per 10 high power (×400) fields. Occasionally, the cell cytoplasm can have few melanin granules [2,28].

##### Cellular Blue Naevus

Tumors are intradermic masses that are non-encapsulated, well-demarcated and symmetrical, composed of fusiform neoplastic cells arranged in interlacing fascicles and supported by collagen fibers. They are localized in the reticular dermis, and extension to the papillary dermis is possible. The overlying epidermis could be demarcated from the tumor by a narrow tumor-free portion on the superficial dermis.

Tumor cells have a clear or eosinophilic cytoplasm with a variable amount of melanin pigment. Nuclei are ovoid and occasionally can have nucleoli (1–2). Their itotic rate averages 1 (range 0–3) mitotic figures per 10 high power (×400) fields. Anisocytosis, anisokaryosis and cellular pleomorphism is mild. It is possible to see some multinucleated tumor cells, which represent degenerative change in naevus. The overlying epidermis may be hyperplastic, hyperpigmented and ulcerated [2,28].

##### Combined Cellular Blue Naevus

The histological appearance is very similar to cellular blue naevus. It is defined as a combination of cellular blue naevus and intradermal common melanocytic naevus due to the presence of tumor cell nests within the basal epidermis. Intraepidermal cells are round and pigmented [2,28].

##### Dermal Melanoma and Dermal Melanomatosis

Dermal melanoma is characterized by round and small heavily pigmented neoplastic melanocytes with a deep dermal or subcutaneous location, whereas dermal melanomatosis refers to multiple and coalescent dermal melanomas. They have variable well-defined margins and sometimes present a pseudo-encapsulation [2,25,28,47].

Tumor cells have variable sizes and pigmentation is often dense, with associated heavily pigmented melanophages also being present. Nuclei are usually large, with a prominent nucleolus. Mitotic figures are more prominent at the margins. Associated changes can include tumor necrosis and intravascular emboli, more frequently in dermal melanomatosis [2,25,28,47].

##### Anaplastic Malignant Melanoma

Anaplastic malignant melanoma is characterized by infiltrative behavior and poorly defined margins, with cells arranged in sheets or cords supported by a fibrovascular stroma. Pigmentation varies largely within the same mass. Intracytoplasmic vacuoles could be present. Nuclei have an irregular shape, may contain multiple nucleoles and are hypochromic. A high mitotic rate is present, with marked pleomorphism. Vascular invasion by tumor cells could be evident, and associated lesions may include epidermal ulceration and lymphoplasmacytic infiltrates [2,25,47].

#### 5.2.3. Immunohistochemistry

Immunohistochemical staining is widely used as complement to histopathology. It helps in the diagnosis of abnormal cells, the differentiation of certain types of tumors and in identifying malignancy when histology alone is not enough. It allows identification of proteins that are associated with carcinogenesis and studies the distribution and localization of biomarkers in different tissues [44,45]. Furthermore, this technique allows the identification of individual cell types within a population of different normal or abnormal cells, for example, the identification of malignant melanotic cells within a regional lymph node. Since prognostic and therapeutics widely differ between types of tumors and between benign and malignant forms, it is important to have a concrete diagnosis [23].

This technique could be of tremendous value in face of a poorly differentiated amelanotic melanoma that may not produce enough melanin to be visible upon routine microscopic evaluation. In this case, a positive immunohistochemical reactivity for melanocytic markers, that cells usually continue to produce, could be used as a diagnostic. Biomarkers currently proposed for equine melanocytic tumor diagnosis are clusters of differentiation 44 (CD44), human melanoma black (HMB-45), Melan-A, S-100 protein, protein gene product (PGP) 9.5, proliferating cell nuclear antigen (PCNA), PNL2, Ki-67 and tyrosinase [46,47,48,49,50,51,52].

According to some authors, HMB45 has significantly higher expression in malignant forms, and CD44 was detected in both benign and malignant forms [47]. S100 protein is a highly sensitive marker but lacks specificity. Many non-melanocytic tumors and normal tissues express this protein, making it unsuitable as an independent test for melanocytic tumor diagnosis [50]. Seltenhammer et al. [45] sustain that PCNA and Ki-67 can be used to differentiate malignant from benign forms.

Recently, Campagne et al. [51] described the overexpression of receptors for activated C kinase 1 (RACK 1) through immunofluorescence. This highlighted the usefulness of RACK 1 as a candidate biomarker for malignant stages, as there was intense, diffuse and homogeneous staining in equine melanoma cells. Data from the cited study report that the heterogeneous cellular distribution pattern of RACK 1 is linked with benign forms, while homogeneity is linked with malign forms. Furthermore, RACK 1 can reflect melanoma progression and aggressiveness, since a homogeneous and diffuse cytoplasmatic labelling of RACK 1 was associated with more aggressive melanoma.

Ramos-Vara et al. [46] aimed to document the immunoreactivity of PNL2, S100 protein, Melan A and PGP 9.5 in fifty melanocytic tumors. All of them expressed PNL2, S100 protein and PGP 9.5. None of the tumors expressed Melan A. None of the normal tissues or non-melanocytic tumors tested expressed PNL2 except the normal melanocytes presented in hair follicles. According to the results, PNL2 was considered a highly sensitive and specific marker for melanocytic tumor diagnosis, with a similar sensitivity when compared to S100 protein and PGP 9.5 but a superior specificity when compared with the same markers. In conclusion, PNL2 can be used alone in the diagnosis of equine melanocytic tumors.

## 6. Differential Diagnosis

Every time there is a black nodular tumefaction, melanocytic tumors should be considered as a potential diagnosis. Examples are nodular sarcoid, cutaneous lymphoma, parasitic cysts, hemangioma, mast cell tumor, fibroma, parotid salivary gland disease and guttural pouch disease, *Gasterophillus* spp. granuloma [2,23]. The absence of black pigment during FNA and the histopathology report of a biopsy sample are the most reliable methods to exclude each of these differentials. However, amelanotic melanomas may raise doubts. In these cases, immunohistochemistry should be used.

## 7. Management

For a long time, the “wait and see” protocol had been the “treatment” of choice, given the benign appearance of the majority of these tumors. Nevertheless, current knowledge easily refutes this approach, with evidence that with time the disease will only get worse clinically and pathologically, decreasing the likelihood of treatment effectiveness [6,7,53].

Before any clinical decision about therapy, some facts and possible limitations should be considered, such as the tumor’s classification, clinical/pathological behavior, location, extent, duration and response to previous treatment attempts, as well as the owner and animal compliance, availability of special equipment and facilities for some treatments and the horse’s purpose [6,7,53].

The effectiveness of surgical treatments is highly dependent on the clinician’s experience, since in some cases the size and location of tumoral mass make it difficult to excise. In most cases, combination of treatments is used and has a better effect than a single method [53]. A graphical review of treatments is shown in Table 3.

### 7.1. Surgical Treatment

**Figure 1:**
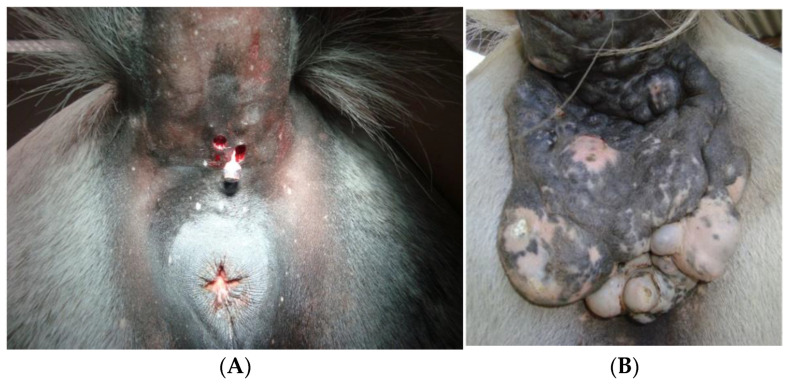
(**A**) Small masses are often easily surgically removable (a biopsy punch is an option for the round and smallest tumors). (**B**) The “wait and see protocol” allows growth and leads to difficult surgical approaches where reconstructive surgery is needed.

Early in the disease process, when most melanocytic tumors are benign and typically have small sizes, surgical excision could be a good option and sometimes curative (Figure 1A). Although some reports suggest that spontaneous regression can occur, the vast majority will expand and eventually ulcerate, become malignant and metastasize. As such, all visible and accessible masses should be removed, limiting the probability of those masses becoming problematic. In the case of melanocytomas, there is no evidence that excision enhances the risk of recurrence or malignant transformation [24,33,53].

In contrast, the more advanced and commonest forms of the disease (dermal melanomas and dermal melanomatosis), are not so easy to manage (Figure 1B). Despite this, surgical excision may be an option in small dermal melanomas or in cases of physiological function disturbance due to tumor growth, such as rectal or perianal obstruction. Coalescent masses can be removed totally or partially to improve the horse’s quality of life, although the procedure is likely challenging and reconstructive surgery could be required [53,54]. In some cases of extensive tumors on the tail, tail amputation could be considered. However, when facing an anaplastic malignant melanoma, even with this aggressive intervention, the outcome is poor [54]. In non-grey horses, wider margins must be taken whenever possible, trying to remove every neoplastic cell, since the likelihood of tumors being malignant and behaving aggressively is bigger than in grey horses. Furthermore, in these cases, histologic examination should always be done [53]. Partial excision may stimulate tumor regrowth and metastatic spread, demonstrating the importance of surgical intervention when total excision can be achieved [53,54]. Groom and Sullins [55] removed large (≥4 cm) single and coalescent melanocytic tumors from 38 horses and followed up the cases for 12 months. Tumor regrowth after surgery was not seen in any case, and no sudden change in size or behavior was noted in the remaining tumors.

Cryosurgery is effective in small, superficial, ulcerated and localized tumors, and better results are achieved when debulking is performed prior to liquid nitrogen application. Some side effects are reported, such as peritumoral tissue damage, pain after surgery, alopecia and depigmentation [54,56].

Carbon dioxide surgery could be an alternative to traditional surgery. This technique allows the diminishing of intraoperative hemorrhage, the elimination of bacteria through generated heat without causing thermal damage, the reduction of postsurgical pain and swelling and a reduction of the risk of tumor cells spreading [57,58].

### 7.2. Chemotherapy

**Figure 2:**
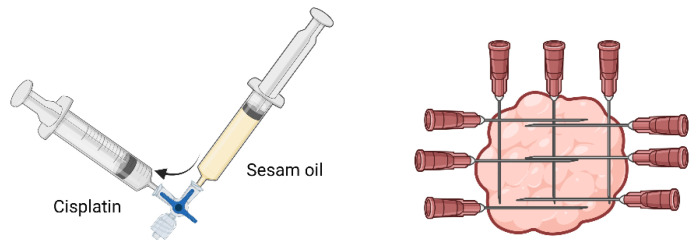
Cisplatin preparation and injection. The tumoral mass should be injected in multiple plans to ensure that cisplatin is equally distributed (created with BioRender.com).

Many treatments have been applied with different success rates [59,60]. Melanocytic tumors, mainly melanomas, are poorly responsive to chemotherapy drugs, needing high concentrations. Cisplatin is one of the few drugs that shows efficacy in some cases, presenting partial remission to complete resolution of some tumors for at least 2 years. Others do not respond at all [60]. Cisplatin toxicity is common when systemically administered. For this reason, intratumoral injection is preferable, with some techniques maximizing local effects and minimizing side effects in normal tissues [61]. Two approaches are recommended: percutaneous injection of a viscous fluid formulation that releases the drug slowly, or surgical implantation of solid polymer matrices loaded with cisplatin. The first one is the most common approach to cutaneous neoplasms [62]. The recommendation for percutaneous injection is 1 milligram of cisplatin (10 mg/mL) per cm^3^ of tumoral mass every two weeks for four treatments. Cisplatin should be mixed with sterile medical-grade sesame oil to hold the substance on the injected spot. Both the tumor and peritumoral region should be injected with this emulsion in multiple plans (Figure 2) [62]. This therapy could be performed in combination with surgical excision, especially in tumors larger than 3 cm in diameter. The disadvantage of this kind of local therapy is that tumor growth in other sites is not prevented [59,62]. The success rate for this type of treatment in equine melanomas has been reported as 81%, with bigger tumors responding less [60,62].

Although more commonly reported in sarcoid and squamous cell carcinoma treatment, Bienert-Zeit et al. [63] reported the use of 5-fluorouracil at the surgical site after melanoma surgical excision to prevent recurrence.

### 7.3. Adjunctive Therapies

**Figure 3:**
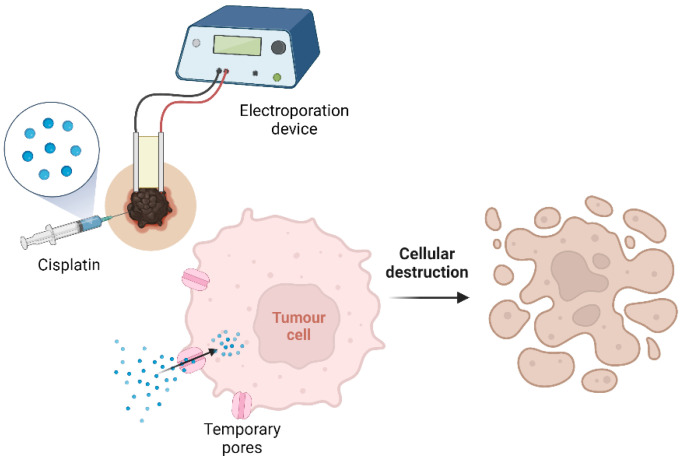
Electrochemotherapy procedure (created with BioRender.com).

Electrochemotherapy is an anticancer therapy that links chemotherapy drugs with electric pulses. It consists of the application of high voltage and low duration electric pulses that result on formation of temporary pores on the cell membrane [64]. Those pores allow drugs to reach the cytoplasm, resulting in cell damage (Figure 3). This technique increases cisplatin uptake and could be applied alone or combined with surgery, increasing electrochemotherapy efficacy [65]. It is also advantageous in anatomical places where a surgical approach is challenging, such as the perianal and eyelid areas.

Currently available, the Chemipulse III portable electroporator (EU patent application number 2221086) allows the treatment of affected horses in the field. Some horses could react to electric pulses, so a high level of sedation is advisable; if the horse does not cooperate or the mass is located in a sensitive area such as the head, general anesthesia is required. In the few existing studies, the results are promising [64]. More studies are needed to spotlight the true outcome of this therapy and eventually assume it as a therapeutic option for these tumors which can be allied to conventional therapies.

Hyperthermia is a therapy on which localized heat is applied in tumor masses alone or in conjunction with chemotherapy. Heat can be generated by ultrasound, radiofrequency, or microwave energy [66]. A commercial thermotherapy unit that is now available (Thermofield System, Parmenides, Inc., London, UK) presents very good results in large tail melanomas. Basically, heat will affect tumor cells in two distinct ways. First, a heat-related stress is created which leads to the production of damage-associated molecular patterns (DAMP’s) with an associated host antitumor immune response. Second, the membranous permeability of tumor cells is enhanced and the cytotoxicity of chemotherapeutic drugs is intensified [6,66]. Some authors suggest that the combination of this technique with chemotherapy has proven highly effective in advanced disease where chemotherapy alone does not take any effect [6].

### 7.4. Calcium Electroporation

Some recent studies have been evaluating this novel anti-cancer treatment. It consists of an intratumoral injection of calcium followed by electric pulses that enhance membrane permeability, allowing calcium uptake [67,68]. Calcium plays an important role in cell metabolism. Intracellular calcium concentration is low (10^−7^ mol/L) and highly regulated, so any alteration affecting this balance can affect cellular processes. The high concentration of calcium after treatment induces cell death [67,68]. An advantage of this method compared with electrochemotherapy is that biosecurity measures are not necessary, as there is no risk of contamination with anticancer drugs. Because of this, the cost of this treatment is inherently lower, which may encourage its widespread use in many veterinary facilities [67,68]. This therapy has not been studied in equine melanocytic tumors yet. However, Galant et al. [69] assessed the effect of intralesional injections of calcium combined with electroporation in 16 horses bearing sarcoid. Treated tumors were microscopically analyzed, with 13 of 16 tumor presenting necrosis with just a single treatment; among the 13 necrotic tumors, nine were necrotic to more than 50%. The only side effect reported was an inflammatory reaction on the injected spot. Frandsen et al. [68] applied this therapy in 27 equine sarcoids, of which six presented total remission and another six presented a decrease in size more than 30%. Given the results, further studies in melanocytic tumors would be interesting.

### 7.5. Irreversible Electroporation

Irreversible electroporation is a novel technique that differs from electrochemotherapy in that electrical pulses irreversibly destroy cell membranes, leading to cell death without the need for chemotherapy. Local tissue heating is low (<5 °C) which allows treatment close to sensible structures such as nerves and vessels. Byron et al. [70] revealed tumor reduction of 52% and 99% after this treatment in two horses with dermal melanomas.

### 7.6. Oral Therapies

Although cimetidine has been reported as a successful treatment on the past, more recent reports do not describe a good outcome for the treated horses [71]. Success apparently appears only in tumors with active growth, with progression of disease being halted over the course of months or years. Doses of 2.5 mg/kg three times daily, 3.5 mg/kg twice daily and 7.5 mg/kg once or twice daily for 6 to 12 weeks are reported. A slow decline in tumor size during the first 6 weeks of treatment was considered a positive response to treatment. If a positive response is noted, daily administration should continue until there is no further improvement. If there are no results in the first 3 or 4 weeks, treatment should be discontinued [32].

### 7.7. Topical Therapy

Betulinic acid (BA) is a natural pentacyclic triterpenoid present in the bark of plane and birch trees. It has demonstrated anti-inflammatory, antiparasitic and anticancer activity [72,73]. This compound, as well as some of its derivatives such as Tris ester NVX-207, have influence in many controlled cell death mechanisms, and their ability to induce apoptosis was demonstrated in an in vitro study using equine melanocytic tumors [74].

Weber et al. [75] presented a recent in vitro study that aimed to evaluate betulinic acid as a topical therapy for melanocytic tumors. For this purpose, a 1% BA in “Basiscreme DAC” with 20% medium-chain triglycerides was used on equine skin and melanoma cells in vitro. Results showed that betulinic acid significantly inhibits cell proliferation and reduces cell viability in a dose and time-dependent manner. Furthermore, it was observed that this formulation was able to pass through the *stratum corneum* and reach the epidermal and dermal layers which cover melanocytic tumors. To support this study, a pre-clinical and clinical trial about the applicability of betulinic acid as a topical treatment for melanocytic tumors would be interesting.

An in vivo test was performed with the same drug formulation previously described as well as with “Basiscreme DAC” containing 1% of NVX-207 on eight healthy horses. The treatment sites were covered with 1 g of the drug formulation twice daily for seven consecutive days. Some treatment sites were covered with wound dressings. The results revealed high local concentrations of BA and NVX-207 after seven days, with the concentration being higher on the covered sites. Furthermore, it was shown that it is safe use both BA and NVX-207, as only mild local adverse effects were reported, such as mild erythema, mild swelling, mild alopecia and mild desquamation. However, since all groups including the placebo had these mild adverse effects, it was assumed that it might be a reaction to the ingredients presented in the carrier cream “Basiscrem DAC” [76].

In another study that included eighteen Lipizzaner horses carrying early stage melanocytic tumors, six horses were topically treated twice daily for 91 days with 1% BA and another six horses with 1% NVX-207, both in “Basiscream DAC”. Wound dressings were applied to completely cover the tumors. Drugs were well tolerated, with the local adverse effects reported being depigmentation of the tumor and overlaying skin and ulceration of the tumor mass. The treated tumors showed a decrease in volume, mainly with BA, since the results of NVX-207 did not reach statistical significance [77].

### 7.8. Radiotherapy

Equine radiotherapy is less common that in other species because of logistical and safety reasons. Nevertheless, an increasing number of specialized facilities are being developed to work with radiotherapy equipment [78].

Teletheraphy and brachytherapy are two modalities of radiation therapy already described. Teletherapy consists of the application of radiation using an external source that is located at some distance from the horse. General anesthesia is required, and multiple treatments need to be done to achieve the prescribed dose. Due to inherent difficulties, few results of this type of therapy are documented [6,58,78]. Brachytherapy is an easier and less costly modality that involves administering radiation within the tumor (interstitial brachytherapy) or adjacent to the tumor (contact radiotherapy). Previously, this technique was performed by sealed radiation implants within the tumor, with iridium being the most common nucleotide. The main disadvantage was that the clinician and owner were exposed to radiation during the treatment time [57].

Electronic brachytherapy is a recent form of interstitial brachytherapy that consists of delivering radiation through a miniature X-ray source. In contrast to the previously mentioned technique, this new one eliminates the risk of handling radioisotopes [6].

Bradley et al. [79] reported successful treatment of a preputial melanoma using the already available Xoft AXXENT^TM^ Electronic Interstitial Brachytherapy System combined with surgical debridement. The best results of brachytherapy are descried in superficial tumors localized in the extremities and without metastatic potential. Furthermore, it is a good option for tumors that do not respond to chemotherapy or that cannot be excised; although, results are better if combined with surgical debridement [80].

### 7.9. Immunotherapy

Melanoma is a tumor with a marked immunogenicity which highlights the potential of responding to immunotherapy [27].

Intratumoral injection of plasmids encoding the cytokines interleukin (IL)-12 and IL-18 was tested in some studies. These cytokines have antitumor effects through the activation of cytotoxic T cells, the production of interferon-gamma and the induction of apoptosis in tumor cells [81]. One study using seven horses and a total of 12 melanomas showed a significant regression in all lesions treated with intratumoral injection of plasmid DNA coding for the human cytokine interleukin 12 [82]. In another study using 26 grey horses bearing melanomas, IL-12-encoding plasmid and IL-18-encoding plasmid were tested, leading to significant size reduction in tumors. The authors suggest that electroporation should be considered to increase efficacy of this therapy [81].

Systemic immunotherapy could be achieved by directing efforts to target tumor-associated antigens, in another words, proteins expressed in tumor tissue. Once identified, immunotherapeutics (also called “cancer vaccines”) can be developed to induce an immune reaction against cells that contain these antigens. The main objective is creating an antitumor immune response that leads to regression of both primary and metastatic tumors [6,83,84]. Only two cancer vaccines have been tested in equine melanoma, an autologous vaccine and a DNA-based vaccine [25,85].

Autologous vaccines are created from excised tumors. Tumoral cells are isolated and then processed in vitro to formulate the vaccine [25]. Since there are still few studies and the presented samples are small, reliable conclusions on their effect cannot be drawn.

DNA vaccine formulation starts with tumor-associated antigen identification. The DNA sequence is cloned on a vector, creating in vivo expression of the protein. The majority of the vector also has immune stimulation capacity, improving the efficacy of the vaccine. This formulation is administered intramuscularly. A free-needle injector device, VitaJet-3 (Bioject Inc., Portland, OR, USA) has been classified as an effective method for intramuscular administration of this vaccine, apparently with less pain and avoiding some risks associated with needle injections [86]. Tyrosinase, a crucial enzyme for melanin production, is a tumor-associated antigen used for DNA vaccine formulation. Its expression is limited to melanocytes, and it presents an overexpression in neoplastic melanocytes compared with normal ones [85]. An available vaccine for the treatment of canine melanoma (Oncept; Merial, Ltd., Athens, GA, USA) encodes human tyrosinase. Once 90% of equine tyrosine sequence shares homology with human tyrosinase, it may be possible to find cross-reactivity [84,85]. A clinical trial presented by Lembcke Perez Prieto in 2013 [87] evaluated a human tyrosinase anti-melanoma vaccine, Oncept^®^ (Merial Limited, Athens, GA, USA), in 10 horses bearing melanomas; the vaccine presented tumor burden reductions and was apparently safe and well tolerated in horses. A suggested dose of 100 μg of tyrosinase plasmid DNA was proposed, the same used in dog melanoma treatments.

Mählmann et al. [88] analyzed the effect of DNA vectors encoding equine (eq) IL-12 and IL-18 vaccine alone or in combination with human glycoprotein (hgp) 100 or human tyrosinase (htyr) in 27 horses bearing melanomas. All groups revealed an average decrease of 79.1% in tumor volume. Cellular and humoral immune responses were not detected specifically against hgp100 and htyr, and the addiction of DNA vectors encoding them did not potentiate the effect of the vaccine.

Finocchiaro et al. [89] reported the use of herpes simplex virus thymidine kinase (HSV*tk*) suicide gene in one horse. Surgical excision of superficial tumors was performed and the margins of surgical wounds were infiltrated by the HSV*tk* suicide gene in conjunction with ganciclovir, since the HSV*tk* suicide gene is reported to increase the sensitivity of transfected cells to this antiviral [88]. The tumors not surgically removed were also infiltrated. The horse was also treated with subcutaneously injected autologous vaccine made from excised tumors. Local recurrence was not observed after surgery, and a significant reduction in size was reported in tumors not surgically removed. No side effects were mentioned.

Although further knowledge is mandatory, with current evidence, one can presume that in the future immunotherapy, alone or combined with other conventional therapies can eventually be part of the cure for this type of equine skin tumor, as assumed by Cavalleri et al. [90].

#### Bacterial Products Injection

Interest in bacteria-based therapy has been reawakened. Aside from identification of bacteria in human cancers that were thought to be sterile, bacterial peptides were identified on the major histocompatibility complex (MHC) of melanoma molecules [90,91]. Thus, this provides a therapeutic target in melanomas for the action of bacteria-specific cytotoxic T cells.

Immune system recognition of pathogen-associated molecular patterns (PAMPs) is the key to the initiation of the immune response. Following this logic, direct injection of bacterial products and therefore of PAMPs into the tumors is able to induce an immune response against the tumor [92].

Carroll et al. [93] performed a clinical trial in humans and animal models by injecting emulsified complete Freud’s adjuvant (CFA) into tumors. CFA is made from mineral oil, surfactant and heat-killed *Mycobacteria*. The slow-releasing formulation injected in the tumor provides continuous immunostimulation for several weeks. Eleven horses carrying naturally occurring melanomas were included and treated with 0.1–0.3 mL of CFA emulsified in saline, injected into their tumors. Reduced mass volume was presented in 3/11 horses, and 2/11 horses showed complete tumor regression.

Data provide evidence that intratumoral injection of CFA is a simple and inexpensive treatment modality for many types of solid tumors, being advantageous both as first-line treatment or after underachievement of other therapies.

### 7.10. Proteasome Inhibition

Over the last decade, many preclinical trials have aimed to evaluate the potential of proteasome inhibition as a treatment for different types of cancer, including melanocytic tumors [94,95].

Amblyomin-X, a Kunitz-type protease inhibitor, is a recombinant protein present on tick *Amblyomma sculptum* salivary glands that has the ability to induce apoptosis and disruption on cells cycle [96,97]. Lichtenstein et al. [96] performed a study to evaluate Amblyomin-X’s effect on four horses. A total of three tumors were treated and the others remained for control. Amblyomin-X was injected intratumorally at 1 mg/kg of tumor mass every 3 days over a period of 28 days. In all cases, a reduction of at least 75% was observed at the end of treatment. Some tumors that belonged to the control group but were close to treated tumors showed some degree of shrinkage. No adverse effects were observed.

## 8. Immunohistochemistry: A Way to New Therapies

Knowledge about carcinogenesis is crucial to direct the research for specific molecular targets [23]. Identifying specific molecules expressed by the tumor predicts a positive response to a certain drug [53]. The lack of information about this topic in equine oncology makes immunohistochemistry a huge and interesting field of research.

CD47 is a transmembrane protein expressed in multiple tumor types and linked to many pathophysiological processes. Anti-CD47 antibodies were previously used to inhibit growth and prevent dissemination, since binding of antibodies to this marker has demonstrated an increase in phagocytosis of cancer cells by macrophages [98]. Caston et al. [99] analyzed the expression of this protein in 24 cutaneous tumors of horses. 54% were positive for CD47 expression, yet the expression on melanocytic tumors was very low despite the low number of samples for this type of tumor (n = 4). Further investigations including larges samples of melanocytic tumors would be useful to provide a more reliable evaluation of CD47 expression on these tumors and elucidate its potential as therapeutic target.

Programmed death-1 (PD-1) is an immunoinhibitory receptor expressed by T-cells and programmed death ligand 1 (PD-L1), also called CD274, is its ligand, being expressed in tumor cells [100]. Interaction between PD-1 and PD-L1 inhibits the effector functions of T cells and the activation signal mediated by T-cells. This suppression regulates excessive immune responses, allowing tumor cells to avoid anti-tumor immune responses and then proliferate and spread [101]. Antibodies targeting PD-1 or PD-L1 have been used to treat many types of human cancers, with very good outcomes reported in advanced human melanomas mentioning long-term tumor regression and prolonged survival [102]. In a recent study, PD-L1 expression was detected through immunohistochemistry in four equine malignant melanocytic tumors, opening a potential novel therapeutical window [103].

Cyclooxygenase 2 (COX-2) is an enzyme present in both normal and tumoral cells. COX-2 has a role in many oncological functions such as immune suppression, inhibition of apoptosis, stimulation of proliferation, cell invasion and angiogenesis [104]. Thamm et al. [105] assessed COX-2 expression in equine tumors, of which 11 were melanocytic tumors. COX-2 was expressed in seven of the 11 melanocytic tumors tested, with 18% presenting a moderate immunoreactivity score and 10% presenting a strong immunoreactivity score. The results demonstrated that melanocytic tumors are prone to react with a good outcome to a COX-2 selective NSAID treatment.

## 9. Conclusions

Although melanocytic tumors have high relevance when it comes to equine skin tumors, much further research is needed due to the lack of knowledge that still exists, mainly when compared with other species.

The high prevalence of grey horses in some breeds increases the need to find successful and easily accessible preventive and therapeutic options.

Research efforts to understand the pathophysiology and biological differences between melanocytic tumors in horses and other species could help to find novel therapeutical targets, and more specific diagnostic and prognostic biomarkers that could help pathologists and clinicians.

## Figures and Tables

**Table 1:** Clinical stages of equine malignant melanoma.

Stage	Number of Masses	Diameter	Growth Pattern	Dissemination
*1*	Single	<2 cm	Slow (same size for months to years)	Absent
*2*	Multiple	<2 cm	Slow (same size for months to years)	Absent
*3*	Multiple	<4 cm	Slow (25% increase in size in months or years)	Present
*4*	Multiple	>4 cm	Rapid (25% increase in size in weeks or months)	Present

**Table 2:** General features of equine melanocytic tumor types.

Tumor Type	Coat Color	Age	Clinical Features	Biological Behavior	Anatomical Localization
**Naevus**	Grey and non-grey horses	Young horses (5–6 yo)	Single masses	Benign	Neck, trunk, limbs and face
**Dermal** **melanoma**	Grey horses	Young horses (6–7 yo) Geriatric	Single or multiple spherical isolated masses	Benign or malignant	Ventral tail, perianal region, external genitalia, lips, eyelids
**Dermal** **melanomatosis**	Grey horses	Geriatric (>15 yo)	Multiple and coalescent masses	Benign or malignant	Ventral tail, perianal region, external genitalia, lips, eyelids
**Anaplastic** **malignant** **melanoma**	Grey horses	Geriatric (>15 yo)	Rare, but with fast growth to multiple and coalescent masses; Heterogeneous color (pink-grey)	Malignant and very aggressive, with high capacity for dissemination; death within months	Unspecified

**Table 3:** Generic compilation of treatment modalities currently used in equine melanocytic tumors.

Treatments								
Surgery	Chemotherapy	Electroporation	Oral	Topic	Radiotherapy	Immunotherapy	Bacterial products	Proteasome inhibition
Traditional surgery Cryosurgery Laser surgery	Cisplatine Adjunctive therapies: Electrochemotherapy Hyperthermia	With calcium Irreversible	Cimetidine	Betulinic acid	Teletherapy Brachytherapy Eletronic brachytherapy	Intratumoral Systemic		Amblyomin X

## Data Availability

Not applicable.
